# Development of a reference material for mercury in fish: certified for total mercury and characterized for methylmercury

**DOI:** 10.1007/s00216-025-05817-z

**Published:** 2025-03-19

**Authors:** Diego A. Garzón, Diego A. Ahumada, Cristhian Paredes, Elianna Castillo

**Affiliations:** 1 Grupo de Estudios Para La Remediación y Mitigación de Impactos Negativos Al Ambiente (G.E.R.M.I.N.A), Departamento de Química, Facultad de Ciencias, Universidad Nacional de Colombia – Sede Bogotá, Carrera 30 # 45-03, 111321 Bogotá, Colombia; 2 Food and Art: Authentication and Sustainability Challenges (FAAST), Department of Chemical Engineering and Analytical Chemistry, Universitat de Barcelona, Martí I Franquès 1-11, 08028 Barcelona, Spain; 3 Grupo de Investigación en Metrología Química y Bioanálisis, Instituto Nacional de Metrología de Colombia, Av Carrera 50 No 26 - 55 Int. 2, Bogotá, D.C Colombia

**Keywords:** Certified reference material, Chemical speciation, Mercury, Methylmercury, Catfish

## Abstract

Reference materials (RMs) and certified reference materials (CRMs) are essential for ensuring compliance of food products with international standards. In fish, mercury content is closely monitored due to associated human health risks. This study developed and certified a CRM for total mercury (Hg) and methylmercury (MeHg) in striped catfish (*Pseudoplatystoma fasciatum*) from the Colombian Amazon. The process involved raw material selection, sample preparation, homogeneity, and stability, resulting in the CRM INM-039–1. Total Hg was measured using inductively coupled plasma-mass spectrometry (ICP-MS) and cold vapor atomic absorption spectrometry (CV-AAS), while MeHg was analyzed by gas chromatography-mass spectrometry (GC–MS). Calibration methods varied: ICP-MS used gravimetric standard addition, CV-AAS employed bracketing calibration, and GC–MS applied matrix-matched calibration. Method validation was performed using CRMs DORM-4 and ERM-CE464. The total Hg results were combined using the Levenson approach, yielding a relative standard uncertainty of 1.8%, while MeHg was 2.9%. Other uncertainty components included long-term stability (2.5%), homogeneity (1.8%), and short-term stability under transport conditions (0.02%). The CRM was certified with a total Hg mass fraction of 3.94 mg/kg (relative expanded uncertainty 7.1%, *k* = 2) and an informative MeHg mass fraction of 3.79 mg/kg (relative expanded uncertainty 8.3%, *k* = 2). This CRM is a valuable tool for assessing mercury in similar matrices, supporting compliance with safety standards for small and mid-size fish producers in the region.

**Graphical Abstract:**

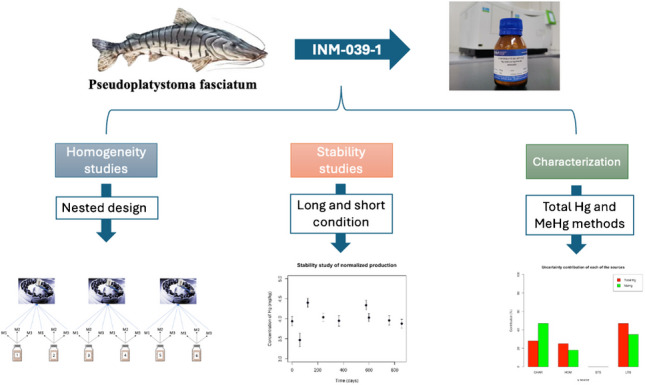

**Supplementary Information:**

The online version contains supplementary material available at 10.1007/s00216-025-05817-z.

## Introduction

The accurate quantification of mercury species in fish is paramount for ensuring food safety, tackling concerns surrounding biomagnification, bioaccumulation, and averting potential adverse health effects in humans [1]. Methylmercury (MeHg) is a highly hazardous form of mercury, prompting regulatory agencies worldwide to establish stringent maximum permissible levels for mercury in fish and shellfish [2]. Despite the Minamata Convention being signed by 128 countries seeking to protect human health and the environment from mercury risks [3], anthropogenic mercury emissions persist. Between 2010 and 2015, mercury emissions to the atmosphere increased by 20%, with South America, sub-Saharan Africa, and Southeast Asia being the main contributors. In South America, approximately 80% of total mercury emissions originate from the Amazon, where the presence of the metal is widespread and highly dynamic [4, 5].

The chemical analysis of mercury species in food matrices requires sophisticated and tailored sample preparation and measurement processes due to the complex nature of the matrix. The process involves extraction, clean-up, and measurement steps [2, 6]. Certified reference materials (CRMs) play a critical role in developing, validating, and assessing the performance of these analytical methods. By ensuring metrological traceability to reference values established through agreed-upon realizations of SI units, CRMs are essential for laboratories seeking accreditation under ISO/IEC 17025 [7, 8].

According to ISO/IEC 17034 and ISO 33405 [8, 9], several approaches are acceptable for the characterization of CRMs. One of the most common strategies for characterizing food reference materials (RM) involves using two or more methods with demonstrable accuracy. When complete characterization is not possible for all analytes, those that meet the requirements can be certified, while the content of the others can be reported as informative values. For an RM to qualify as a CRM, at least one of the property values must be certified with an associated uncertainty and traceability to SI units.

Some national metrology institutes (NMIs) have developed CRMs to support the determinations of mercury and its species in fish. Notably, the National Research Council of Canada (NRC) offers the DORM series [10], and DOLT series [11] CRMs, The National Institute of Standards and Technology from the United States (NIST) offers the SRM 1566b [12], while the Institute for Reference Materials and Measurements (IRMM) used to provide ERM-CE464 [13], which is currently out of stock. However, the certified contents of mercury in the currently available CRMs cover low mercury concentrations, ranging from 0.04 to 0.44 mg/kg, which does not adequately capture the high mercury levels often reported in Latin American fish [14]. This gap reflects an absence of CRMs tailored for the river fish species typical of the Latin American region, which might contain of mercury levels. The striped catfish (*Pseudoplatystoma fasciatum*) is one of the most consumed in the Amazonian region [15–18]. Therefore, a CRM with higher levels of mercury is needed for the quality assurance of the mercury measurements in the Latin American context.

Since the current CRMs do not adequately address the analytical complexities prevalent in Latin America, where mercury concentrations in fish often exceed those reflected in existing CRMs [17], this study aimed to develop a CRM fitted for Latin American river fish species, particularly the striped catfish, addressing the unique analytical challenges posed by elevated mercury levels. Additionally, it includes a comparative analysis of two batches of reference materials derived from fish of varying sizes.

## Materials and methods

### Collection of samples and preparation of the reference material

To generate a reference material for mercury in striped catfish, two production batches of reference materials were undertaken. The initial batch consisted of a pilot production, focusing on establishing and validating the preparation procedure, assessing homogeneity, and conducting stability studies, to facilitate subsequent productions. After the pilot production, a normalized production was executed to supply a comparison item for proficiency tests, inter-laboratory comparisons, and later commercialization at the National Metrology Institute of Colombia INM.

#### Pilot production

For the pilot production**,** a single 34-kg specimen of the *Pseudoplatystoma magdaleniatum* fish was purchased dead from a local market in the Amazonas region without tail and head. The fish, devoid of head or tail, was filleted to remove the skin and bones. Post-filleting, the fish muscle tissue was retrieved, frozen at − 80 °C, and freeze-dried at 0.5 mBar and − 15 °C. The lyophilized tissue was defatted via hexane extraction as per AOAC method 960.39 [18], followed by drying in a vacuum oven. The dry, degreased tissue was grinded and sieved. The fraction between 118 µm and 230 µm was thoroughly mixed, aliquoted, and packaged in amber glass bottles containing 15 g of material. The pilot production yielded a batch of 76 units that were sterilized using gamma rays (^60^Co, 20 kGy).

#### Normalized production

During the pilot production, the processed fish tissue from the large specimen was found to have a mercury content nearing 20 mg/kg; hence, the normalized production was proposed using smaller specimens with reduced mercury levels [19]. Five specimens of striped catfish, approximately 7 kg each, from Leticia (Colombia), were used for this purpose. After filleting, skin removal, and bone extraction, the fish muscle tissue underwent freeze-drying, degreasing, grinding, homogenization, aliquoting and packaging, and sterilization, repeating the steps of the pilot production. The normalized production yielded 119 bottles with 15 g of the CRM candidate. After packaging, the CRM was measured for water activity (4TE Water Activity Meter, Aqualab, USA), total fat, using the AOAC 960.39 method [20], and microorganisms content. The latter was assessed by culturing aerobes, mesophiles, fungi, and yeasts on Potato Dextrose Agar (PDA) and Violet Red Bile Agar (VRBA).

The Hg and MeHg content reported in this work was determined on a dry basis. In each case, a 0.5 g portion of the material was used for moisture analysis, which was dried at 70 °C in 2-h intervals until a constant weight was achieved. This sample portion was independent of the one used for measurement The sample portion in all experiments (homogeneity, stability, and characterization) was 0.5 g of the produced fish material.

### Total mercury determination

Each subsample (0.5 g) was treated with 3 mL of concentrated, doubly subdistilled nitric acid and 2 mL of 30% ultrapure-grade hydrogen peroxide (Merk, Germany). To stabilize mercury in the solution, 0.2 mL of a 100 µg/kg gold ion solution (SMU, Slovakia) was added [21]. The samples were then digested in a microwave oven (Multiwave 5000, Anton Paar, Austria) under the following conditions: an initial temperature of 145 °C for 15 min, followed by a final temperature of 190 °C for 20 min, and a cooldown phase to 20 °C. After digestion, the samples were allowed to cool and rest for 12 h to prevent mercury losses. Subsequently, the extracts were diluted to a final weight of 20 g with deionized water (0.055 µS/cm, Elga, Purelab Flex, UK), and the mercury content was determined using inductively coupled plasma mass spectrometry (ICP-MS) and cold vapor atomic absorption spectrometry (CV-AAS).

#### ICP-MS analysis

The ICP-MS (NexION300D, Perkin Elmer, MA, USA) was operated with the following parameters: RF power of 1400 W, a cooling gas flow rate of 13.5 L/min, an auxiliary argon gas flow rate of 0.82 L/min, a sample argon gas flow rate of 1.42 L/min, a dwell time of 30 ms, and 60 sweeps. Isotope monitoring included ^74^Ge, ^199^Hg, ^200^Hg, ^201^Hg, and ^202^Hg. Germanium (Ge) was used as the internal standard, selected after evaluating various alternatives (e.g., Tl, Bi). The auxiliary gas flow rate was set to 0.70 L/min, while all other conditions remained as described. The measurement sequence was randomized, and digestion blanks, along with the certified reference material (CRM) ERM-CE 464, were included for quality control.

#### CV-AAS analysis

During the characterization of the candidate reference materials, a second method was also employed: cold vapor atomic absorption spectrometry (CV-AAS). This method was performed using a PinAAcle 900 T system coupled to a FIAS 400 module (Perkin Elmer, MA, USA). For CV-AAS, a hollow cathode lamp (HCL) with a detection wavelength of 253 nm was employed. Argon was used as the carrier gas at a flow rate of 50–100 mL/min, with a cell temperature of 100 °C and a 500 µL sample loop.

### Methyl mercury determination

The determination of methylmercury (MeHg) was performed using gas chromatography-mass spectrometry (GC–MS) on a Clarus 690 system equipped with an SQ8C mass spectrometer (Perkin Elmer, Massachusetts, USA). The separation was achieved using a 5% diphenyl/95% dimethyl polysiloxane column (Elite 5, 30 m length, 250 µm inner diameter, and 0.25 µm film thickness). The temperature program began at 45 °C, held for 3 min, and increased at a rate of 10 °C/min until reaching 240 °C, where it was held for an additional 3 min. Helium was used as the carrier gas at a flow rate of 2.2 mL/min. The transfer line temperature was set at 280 °C, with an electron impact energy of 70 eV and a source ionization temperature of 180 °C. Ion monitoring included the following masses: 260, 246, 202, 201, and 198.

The sample preparation for MeHg determination involved extraction with tetramethylammonium hydroxide (TMAH, in 25% KOH) in a thermostatic bath at 80 °C for 8 h. The extracted MeHg was then derivatized using 2% sodium tetraethyl borate, followed by extraction of the derivatized product with a 9:1 mixture of toluene and isooctane. This method was adapted from the procedure described by Gajdosechova [22]. The bracketing method, with internal standard correction, was applied using an isotope-enriched compound, ((Et)_2_^202^Hg).

For quality control, the certified reference materials (CRMs) DORM-4 and ERM-CE 464 were analyzed alongside the samples. The SRM® 3133 from the National Institute of Standards and Technology (NIST) was used as the calibrant for total mercury determinations. For MeHg measurements, a derivatized MeHgCl solution at 1000 mg/kg was employed, with its mercury content quantified using SRM® 3133. These quality control measures ensured the accuracy and reliability of the MeHg determinations.

### Homogeneity studies

The homogeneity of the candidate reference materials was evaluated using a nested design, analyzing total mercury by ICP-MS (“Collection of samples and preparation of the reference material” section). For this study, the most abundant isotope was used, as it provided the best precision. Two experiments were conducted during the pilot production phase. The first experiment aimed to identify the mercury and internal standard isotopes that optimized measurement performance, while the second focused on estimating the uncertainty associated with homogeneity. In the first experiment, one bottle was analyzed with seven subsamples, whereas in the second, 12 bottles were analyzed with one subsample each. For the normalized production, a nested design was also implemented, involving 12 units and seven subsamples per unit.

Statistical treatment for uncertainty determination employed one-way ANOVA for pilot production and a two-way ANOVA for the normalized production, utilizing Eq. 1 where u_hom_ represents the variance between bottles, MS_between_ denotes the between bottles mean square, MS_residual_ indicates the mean square of residuals, and n_0_ signifies the number of observations [20].

1 $${\text{u}}_{\text{hom}}^{2}=\left(\frac{{\text{MS}}_{\text{between}}-{\text{MS}}_{\text{residual}}}{{\text{n}}_{0}}\right)$$

### Stability studies

The stability of the produced reference material for mercury content determination was evaluated through isochronous and classic studies, following ISO 33405 Sect. 8.2 [9]. Two distinct conditions were investigated to assess their impact on mercury stability; for all conditions, the reference condition was − 80 °C. In the isochronous design, short-term stability (STS) and long-term stability (LTS) were assessed in the pilot production, while in the classic design, LTS was assessed in the normalized production.

The STS evaluated potential changes during CRM transportation by placing the samples at 60 °C and 80% relative humidity for 1 month, with weekly measurements to monitor changes in mercury content. LTS was conducted to predict potential changes in the CRM over its shelf life; samples were stored at 20 °C, and two bottles were sampled approximately every 6 months over a 24-month period. The same analytical methods and conditions used in the homogeneity studies were applied.

Linear regression analysis was employed to determine the kinetic constants for each storage condition. These constants were then integrated into Eq. 2 (STS) or Eq. 3 (LTS) to estimate the uncertainties associated with short-term (u_sts_) and long-term (u_lts_) stability.

2 $${\text{u}}_{\text{sts}}={k}_{st} ({\text{t}}_{\text{st}})$$

3 $${\text{u}}_{\text{lts}}={k}_{lt} ({\text{t}}_{\text{m}1}+{\text{t}}_{\text{cert}})$$

where t_st_ is the time for the short-term stability study, t_m1_ is the study duration (730 days), t_cert_ is the material validity time, and ${k}_{st}$ and ${k}_{lt}$ are the calculated kinetic constants for the short-term and long-term studies, respectively [9].

### Characterization: measurement of the material

The property values measured in the candidate reference materials were the mass fractions of total mercury (Hg) and methylmercury (MeHg). The analysis of total Hg was performed using two complementary methods, both employing the digestion procedure described in the “Collection of samples and preparation of the reference material” section. For each method, a minimum of three bottles were analyzed under intermediate precision conditions (i.e., on different days). On each measurement day, digestion or extraction was performed on at least seven subsamples. Subsequently, all data were processed to obtain a single concentration value for each technique.

To combine the measurement results from the two techniques, the Levenson approximation was applied [19]. Uncertainty estimation was made according to the Guide to the Expression of Uncertainty in Measurement (GUM) [23].

The details of the validation process and the most relevant results obtained for each method are provided in the Supplementary Material. This document provides detailed information on the validation process, including selectivity, linearity, trueness, precision, and uncertainty estimation.

### Metrological traceability

All reference materials used in this study were certified and produced by recognized metrology institutes, including the National Research Council of Canada (NRC), the Slovak Institute of Metrology (SMU, Slovakia), and the National Institute of Standards and Technology (NIST, United States), among others. These materials ensured the traceability and reliability of the measurements performed.

All weighing operations, preparation of solutions and reagents, dilution of certified reference materials (CRMs), and other procedures essential for the implementation of the methods in this study were conducted gravimetrically. A Mettler Toledo XPE 205 analytical balance, with a resolution of 0.01 mg, was used for these operations. The balance was calibrated with an E2-weight set, ensuring traceability to the International System of Units (SI).

## Results and discussion

### Preparation of the material of reference

The preparation of the reference material involved two distinct production batches: the pilot and the normalized. A comparative analysis between these two batches provides insights into the optimization of production processes and the resulting material characteristics.

The pilot production utilized a single large fish weighing 34 kg. Despite efforts to optimize the process, the yield obtained of processed material to raw material was relatively low at 4%. Filleting and deboning incurred the highest losses, while freeze-drying and degreasing also contributed to significant material loss. Moisture content was 4%, with a water activity of 0.14, and no microbial presence was detected [16, 17].

In contrast, the normalized production involved smaller fish sizes (7 kg), resulting in a significantly higher yield of 10%. This increased yield was attributed to a more efficient filleting and deboning process, leading to greater lyophilized muscle yield. The moisture content after freeze-drying was reduced to 3.2%, indicating improved process efficiency. The lower freeze-drying temperature (− 25 °C compared to − 15 °C in the pilot production) facilitated sublimation and enhanced performance. The water activity of the material produced in the normalized production was 0.23. Additionally, the fat content analysis revealed a percentage of 3.4%, indicative of a stable and durable material [14, 24]. During the initial study, different fractions were collected during sieving, and homogeneity was evaluated for each. The 118–230 μm fraction provided the best balance between quantity and homogeneity, consistent with previous studies, and was selected for further characterization and production [13, 14].

**Fig. 1:**
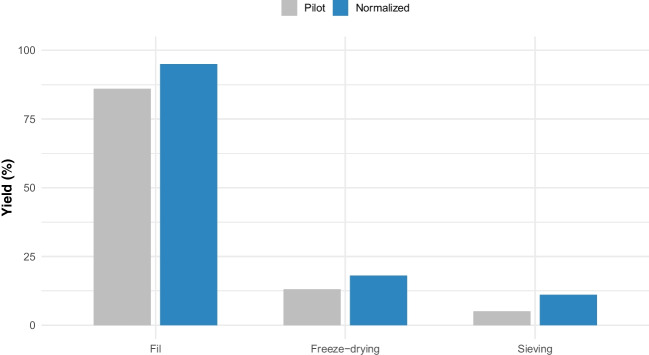
Comparison of yields and process efficiency between pilot and normalized production batches

The yields estimated for the two materials (pilot and normalized) at three different stages of the process (filleting, freeze-drying, and sieving) are illustrated in Fig. 1. Overall, the normalized production exhibited superior performance compared to the pilot production in terms of yield. The optimization of production processes in the normalized production batch resulted in higher efficiency and improved material characteristics. These findings underscore the importance of process optimization in the production of reference materials, contributing to their reliability and suitability for analytical applications.

### Homogeneity

Before estimating the homogeneity uncertainty for the two batches produced, trends in packaging and measurement were evaluated. Linear regression analysis revealed no significant trends (*p* > 0.05) in either case. Following this, uncertainty was quantified using analysis of variance (ANOVA) in accordance with ISO Guide 33,405, ensuring a reliable estimation of variability [20].

**Fig. 2:**
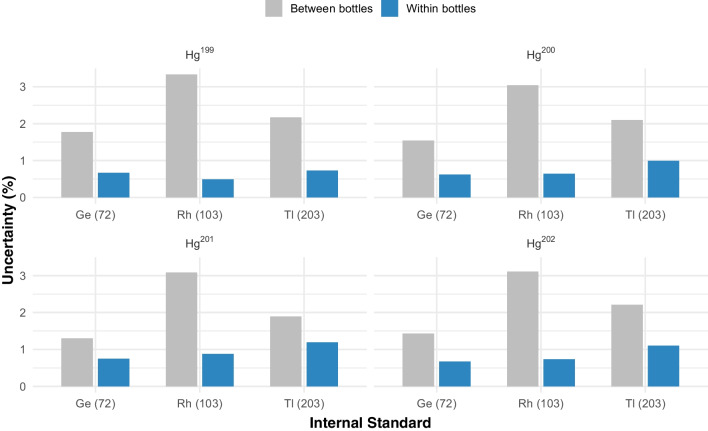
Within-bottle homogeneity uncertainties estimated in the mercury in fishmeal pilot material homogeneity study

Figure 2 illustrates the uncertainties due to homogeneity determined in the study for different combinations of mercury isotopes and internal standards, depicting within-bottle and between-bottle variation. Analysis revealed variations in uncertainty estimation for different combinations of mercury isotopes and internal standards. Ge emerged as the preferred internal standard, exhibiting lower uncertainties for most Hg isotopes. This conclusion was supported by smaller mean squares of error, indicating better uncertainties with this internal standard combination. The relative uncertainty of the pilot CRM was 3.1%.

Regarding the normalized production, non-significant trends were observed in both measurement and packaging for the subsequent batch. The homogeneity uncertainty for this batch was 1.8%, lower than that of the pilot production (3.1%). This reduction is attributed to improved measurement conditions, leading to a more accurate estimation of variation between bottles.

Finally, when comparing the normalized material with other mercury in fish, several valuable insights emerge. Regarding DORM-4, which has a concentration of 0.410 mg/kg and an uncertainty of 3.80%, its significantly lower concentration warrants caution in making direct comparisons due to the substantial difference in concentration. In contrast, when comparing the normalized material with ERM-CE 464 (concentration: 5.24 mg/kg, homogeneity uncertainty: 3.90%) [13], a similar total mercury concentration is evident; however, the normalized material has lower homogeneity uncertainty of 1.80%. Furthermore, the ERM-CE 464, offering a promising reference for accurate mercury analysis.

### Short-term stability

**Fig. 3:**
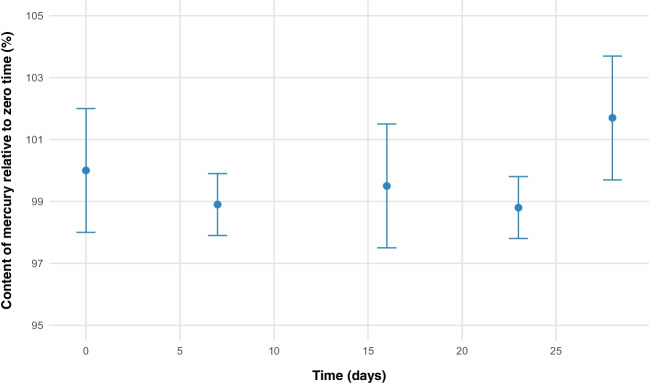
Short-term stability for mercury (^201^Hg/^74^Ge) in striped catfish pilot production

The results obtained from the short-term stability study using an isochronous design conducted over 28 days at 60 °C and 80% relative humidity in the pilot material are presented in Fig. 3. The figure displays the relative to the day zero of the study.

To evaluate changes in the mercury mass fraction over time and determine the best kinetic model for degradation, different kinetic orders were tested. To estimate the uncertainty attributable to the short-term stability of the material a zero-order kinetic model was utilized. The relative uncertainty due to short-term stability is estimated for the pilot material as 0.02% at 30 days under temperature and moisture conditions of 60 °C and 80%.

On the other hand, in the short-term stability assessment of the normalized batch, the results showed an identical behavior in its stability; uncertainty was re-evaluated using the standard deviation of the slope within the framework of a zero-order kinetic model. The quantified uncertainty was 0.02%, demonstrating that the preparation process could be reliably reproduced across different batches and the stability is the same.

### Long-term stability

**Fig. 4:**
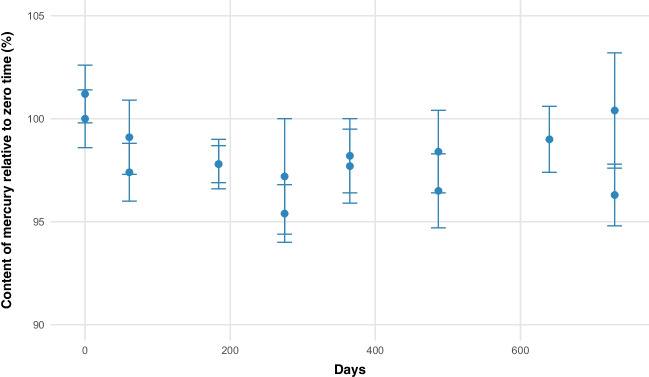
Long-term stability for mercury (^201^Hg/^74^Ge) in striped catfish in pilot material

The long-term stability study was conducted for the pilot batch over 730 days, during which total mercury measurements were taken at temperatures ranging from 15 to 21 °C and a relative humidity of 40% ± 16%. Figure 4 presents the results obtained in the determination of mercury at different times in the pilot batch.

A simple linear regression analysis indicated a significant trend in mercury concentration over the study period when considering all sampling points. Different kinetic models were assessed to determine the best fit (supplementary material), but zero kinetic order was used for uncertainty estimation because it is the most used model. The uncertainty due to long-term stability was 2.5%.

Comparison with similar reference materials, such as ERM-CE464 and DORM-4, revealed that mercury reference materials in fish may have a shelf life of up to 10 years. The findings of this study are consistent with these observations, demonstrating that the reference material maintained adequate stability over the 2 years. It is likely that with further studies conducted over a more extended duration, the material could remain valid for an even longer period [13, 25].

### Stability of the normalized CRM

**Fig. 5:**
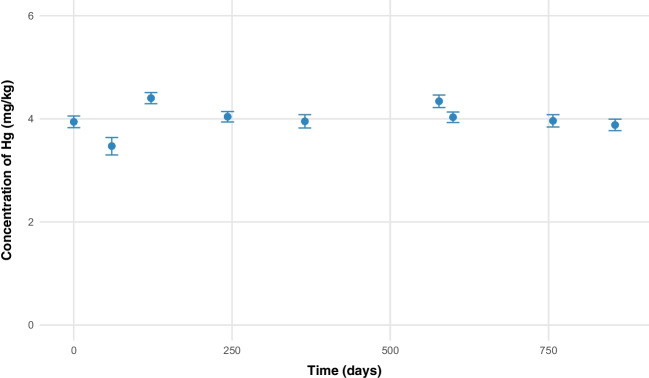
Long-term stability for mercury (^201^Hg/^74^Ge) in striped catfish in normalized material using a classical design

To determine whether the behavior of the pilot material is comparable to that of the standardized material, a stability study was conducted using a classic design over 855 days. Figure 5 presents the results obtained. In this study, no significant slope was observed (*p*-value of 0.74), indicating that no instabilities occurred in the material over the entire period.

### Characterization of the material

The pilot CRM was to assess the preparation conditions and conduct long-term studies, and the characterization process was not conducted for this batch; total Hg concentration was estimated using CV-AAS method obtained a value of 20.88 mg/kg and an RSD of 1.72% with *n* = 5.

The results presented below pertain to the normalized CRM batch. The characterization of total mercury in the material utilized two analytical techniques with demonstrated accuracy. During the SAM-ICPMS method, four isotopes of Hg (^199^Hg, ^200^Hg, ^201^Hg, and ^202^Hg) were considered and the variations between these values contributed to uncertainty due to the combination of the measured isotopes (between isotopes bias). The between-isotope bias was estimated at 0.14%, while the standard relative uncertainty of the method was 2.6%. The mathematical model used and the estimation of uncertainty for this method are provided in the supplementary material. In summary, the first-day measurement yielded 4.00 ± 0.2 mg/kg, and the second day was 3.99 ± 0.07 mg/kg. The mean and combined uncertainty were 4.00 ± 0.11 mg/kg (2.63% as relative uncertainty) obtained by conventional average approximation. ERM-CE464 served as quality control for the measurements, yielding a non-statistically significant of 1.0%.

The second method, CV-AAS with external bracketing calibration, showed stable measurements over 3 days. The mean value was 3.87 ± 0.079 mg/kg (2.0% as relative uncertainty). When comparing the two methods, CV-AAS exhibited lower uncertainty, which can be attributed to its selectivity, minimal matrix effect, and memory effect [26, 27]. These values were employed in the value assignment process presented in Table 1.

**Table 1:** Combination of methods for assigning total Hg value

Method	Concentration (mg/kg)	Standard uncertainty (mg/kg)
SAM-ICPMS	4.002	0.11
CV-AAS	3.873	0.079
Mean (mg/kg)		3.937
between methods uncertainty (mg/kg)		0.037
within method uncertainty (mg/kg)		0.065
Estimated uncertainty (mg/kg)		0.071
Relative standard uncertainty		1.91%

The measurement of methylmercury was exclusively performed using GCMS, and therefore, this value was not certified. The analysis involved two independent measurement days, each comprising five subsamples and five instrumental replicates for each subsample. To ensure accuracy, CRM DORM-4 served as a control. The mean measurement value for MeHg in the fish material was 3.79 ± 0.11 mg/kg (2.9% relative standard uncertainty). On the first day, the value was 3.82 ± 0.11 mg/kg, and on the second day, it was 3.77 ± 0.19 mg/kg. Notably, the maximum bias observed during this measurement for the DORM-4 was 3.3%, which is not statistically significant using as a criterion the combination of uncertainties of the MRC DORM-4 and measurement method with a coverage factor of 2.

### Certified value

**Fig. 6:**
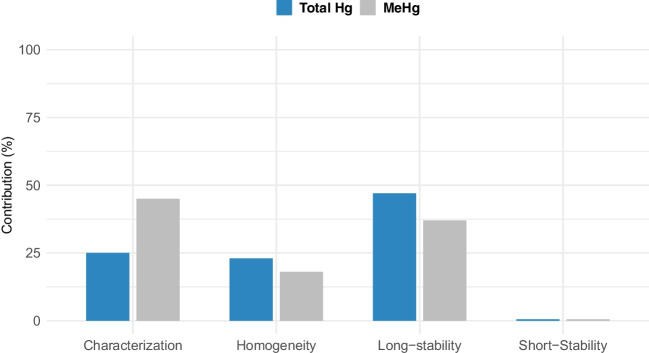
Contribution of uncertainty sources to the value assignment of INM-039–1. The sources include property value measurement (characterization), batch homogeneity (homogeneity), short-term stability (short-stability), and long-term stability (long-stability)

The assigned values for total mercury and methylmercury were determined by considering all previously evaluated sources of uncertainty. For total mercury, the certified value was 3.937 ± 0.27 mg/kg (*k* = 1.97, 95% level of confidence), while for methylmercury, the informative value was 3.79 ± 0.31 mg/kg (*k* = 1.97, 95% level of confidence). The uncertainty contribution of each of the sources shown in Eq. 1 is shown in Fig. 6.

The developed CRM exhibits comparable total mercury and methylmercury concentrations to ERM-CE464 [13], which has concentrations of 5.24 ± 0.10 mg/kg and 5.50 ± 0.17 mg/kg, respectively. Additionally, in terms of stability, homogeneity, and particle size, it is also comparable to other similar CRMs used in this type of analysis, such as NRC’s DOLT 4 and DORM 4.

## Conclusions

In this study, two batches of reference materials were successfully prepared, providing critical insights into their preparation, homogeneity, and stability. Utilizing a single fish specimen to obtain a pilot batch resulted in notably high mercury concentrations (20 mg/kg); however, the yield of processed material from raw material was only 4%. Conversely, the normalized batch, composed of smaller specimens with lower fat content, achieved yields of up to 10% and lower mercury concentrations, approximately 4 mg/kg. Homogeneity studies revealed uncertainties consistent with the intended purpose. Additionally, stability analyses demonstrated that both batches maintained comparable stability levels, allowing for the certification of mercury content for up to 2 years.

A mercury CRM in fishmeal was prepared and characterized with a total mercury concentration of 3.937 mg/kg, featuring a relative expanded uncertainty of 7.1% (k = 2, 97% confidence level). The methylmercury concentration was 3.795 mg/kg, with a relative expanded uncertainty of 8.3% (k = 2, 98% confidence level). The material is valid for more than two years under storage conditions and can withstand up to 30 days in transport conditions. The main sources of uncertainty identified during production were property value and long-term stability. The developed material is comparable to the reference material produced by the European Commission JRC (ERM-CE464), with an inorganic mercury value of 5.24 mg/kg ± 0.10 mg/kg and an organic mercury value of 5.50 mg/kg ± 0.17 mg/kg, given the similar concentrations. Regarding other characteristics such as stability, homogeneity, and particle size, it is also comparable to certified materials like NRC DOLT 4 and DORM 4. Additionally, this work emphasizes the strong need to build scientific evidence to assess the impact of mercury contamination on human health at regional scales.

## Supplementary Information

Below is the link to the electronic supplementary material.

Supplementary file1 (DOCX 27 KB)
