# Supplementary material for: Development of a reference material for mercury in fish: certified for total mercury and characterized for methylmercury
Source: Anal Bioanal Chem. 2025 Mar 19;417(12):2717–26. doi: 10.1007/s00216-025-05817-z (PMC12003448; doi:10.1007/s00216-025-05817-z)
Supplement: Supplementary file 1 — Supplementary file1 (DOCX 27 KB) [file 216_2025_5817_MOESM1_ESM.docx]

**Supplementary Material 1: Method validation**

**DEVELOPMENT OF A REFERENCE MATERIAL FOR MERCURY IN FISH: CERTIFIED FOR TOTAL MERCURY AND CHARACTERIZED FOR METHYLMERCURY**

**Diego A. Garzón^1,2,^, Diego A. Ahumada^2,3^, Cristhian Paredes^2^ and Elianna Castillo^1*^**

^1^Grupo de Estudios para la Remediación y Mitigación de Impactos Negativos al Ambiente (G.E.R.M.I.N.A), Departamento de Química, Facultad de Ciencias, Universidad Nacional de Colombia – Sede Bogotá, Carrera 30 # 45-03, Bogotá 111321, Colombia

^2^ Food and Art: Authentication and Sustainability Challenges (FAAST), Department of Chemical Engineering and Analytical Chemistry, Universitat de Barcelona, Martí i Franquès 1-11, E08028 Barcelona, Spain

^3^Grupo de Investigación en Metrología Química y Bioanálisis, Instituto Nacional de Metrología de Colombia, Av Carrera 50 No 26 - 55 Int. 2., Bogotá, D.C., Colombia

*Corresponding author. e-mail: [ecastillo@unal.edu.co](mailto:ecastillo@unal.edu.co)

To ensure the reliability of the results, three analytical methods were validated: (i) ICP-MS for total mercury determination, (ii) CV-AAS for total mercury determination, and (iii) GC-MS for methylmercury determination. Section 1.1 describes the validation process, including the criteria and procedures used to evaluate the performance of the methods. Section 1.2 presents the most relevant results obtained during the validation, highlighting the suitability of the methods for their intended purpose. Finally, Section 1.3 describes the general approach used for uncertainty estimation, detailing the steps and considerations involved in the process.

## Method Validation

The performance of the methods was evaluated based on selectivity, linearity, bias (trueness), and precision under repeatability and inter-day conditions. For this purpose, real catfish samples and certified reference materials (ERM-464, DORM-4, and DOLT-5) were analyzed. Linearity was assessed using four statistical tests: (i) regression ANOVA, (ii) intercept significance test, (iii) slope significance test, and (iv) lack-of-fit ANOVA test. Selectivity was evaluated by ensuring that the signal-to-noise ratio was less than 5% of the signal obtained at the first level of the determined linear range. Bias and precision were assessed using the normalized error and the Horwitz coefficient of variation as estimators. Finally, the uncertainty of each method was estimated following the Guide to the Expression of Uncertainty in Measurement (GUM). As a criterion for method suitability, the measurement uncertainty was required to be less than one-third of the target uncertainty of the reference material (12%).

## Results of method validation

The three methods used to quantify Hg and MeHg in the certified reference material (CRM) were validated as follows:

- **ICP-MS for Total Hg Determination:**The method for determining total Hg using ICP-MS demonstrated an instrumental linear range from 15 µg/kg to 125 µg/kg. The signal-to-noise ratio at a signal level of 15 µg/kg was 5%. Repeatability was 2.8%, and the normalized error obtained for the CRMs used in accuracy testing met the criterion established by ISO 33. The measurement uncertainty of this method was 2.0%, with the primary source of uncertainty being the method's precision, which contributed 82% to the combined uncertainty.
- **CV-AAS for Total Hg Determination:**The CV-AAS method for total Hg determination exhibited performance parameters comparable to those of ICP-MS. Its linear range extended from 1 µg/kg to 50 µg/kg, and its suitability was confirmed using the statistical tests described in the methodology section. Selectivity was verified, with the signal-to-noise ratio being 2% of the signal at 1 µg/kg. Precision under repeatability conditions was 3%, and the normalized error was below the established criterion. The standard measurement uncertainty for this method was 1.6%, with 75% of this value attributed to precision.
- **GC-MS for MeHg Determination:** The GC-MS method for MeHg determination demonstrated a linear range from 50 µg/kg to 1500 µg/kg, with a signal-to-noise ratio of 1% at 50 µg/kg. The repeatability of the method was 5.5%, and the normalized error complied with the criterion established by ISO 33. The estimated measurement uncertainty for this method was 3.6%, which was higher than that of the total Hg determination methods, primarily due to the nature of the measurement process. As with the other methods, the largest source of uncertainty was the method's precision, contributing 64% to the combined measurement uncertainty.

## Uncertainty Estimation

The estimation of uncertainty for all methods was conducted following the guidelines provided in the Guide to the Expression of Uncertainty in Measurement (GUM).

For all methods, the uncertainty estimation considered, at a minimum, the following factors: sample weighing, regression models (where applicable), covariances, weighing operations, calibration certificates of balances, and certified reference materials, among others. All potential sources of uncertainty associated with the methods were included in the analysis, without discarding any contributions. For instance, in the case of weighing, factors such as air buoyancy were explicitly accounted for.

The combination of uncertainties was performed using the measurement model proposed for each method, applying the analytical solution to derive the combined uncertainty. Additionally, the contributions of individual uncertainty sources were analyzed to identify opportunities for improvement, thereby enhancing the overall reliability of the characterization process.

It is important to note that all uncertainty estimations were carried out prior to the characterization of the reference materials. This ensured that the methods were fully validated and optimized before their application in the study.

SAM-ICPMS Method.

- Model quantification

$$\boldsymbol{C}_{\boldsymbol{m}}=\frac{\boldsymbol{C}_{\boldsymbol{n}\mathbf{2}}*\left( \boldsymbol{R}_{\boldsymbol{m}}-\boldsymbol{R}_{\boldsymbol{n}\mathbf{1}} \right)-\boldsymbol{C}_{\boldsymbol{n}\mathbf{1}}\left( \boldsymbol{R}_{\boldsymbol{m}}-\boldsymbol{R}_{\boldsymbol{n}\mathbf{2}} \right)}{\boldsymbol{R}_{\boldsymbol{n}\mathbf{2}}-\boldsymbol{R}_{\boldsymbol{n}\mathbf{1}}}*\boldsymbol{FD}_{\boldsymbol{m}}*\boldsymbol{FD}_{\boldsymbol{d}}*\boldsymbol{R} (\mathbf{1})$$

- Uncertainty estimation

| Source | Value | Standard_Unit | Unit | Sensitivity_Coefficient | Contribution |
| --- | --- | --- | --- | --- | --- |
| Estimated Concentration | 0.0591 | 4.74E-04 | mg/kg | 67 N/A | 17.73% |
| δm | NA | 4.04E-05 | NA | 4 mg/kg | 0.00% |
| δf | NA | 5.77E-05 | NA | 4 mg/kg | 0.00% |
| δEI | NA | 5.77E-05 | NA | 4 mg/kg | 0.00% |
| Dilution by Digestion (FDD) | 66.83 | 8.87E-03 | NA | 0.059 mg/kg | 0.00% |
| Repeatability | NA | 0.0181 | mg/kg | 4 N/A | 82.26% |

CV-AAS Method.

- Model quantification

$$\boldsymbol{C}_{\boldsymbol{m}}=\frac{\boldsymbol{C}_{\boldsymbol{n}\mathbf{2}}*\left( \boldsymbol{R}_{\boldsymbol{m}}-\boldsymbol{R}_{\boldsymbol{n}\mathbf{1}} \right)-\boldsymbol{C}_{\boldsymbol{n}\mathbf{1}}\left( \boldsymbol{R}_{\boldsymbol{m}}-\boldsymbol{R}_{\boldsymbol{n}\mathbf{2}} \right)}{\boldsymbol{R}_{\boldsymbol{n}\mathbf{2}}-\boldsymbol{R}_{\boldsymbol{n}\mathbf{1}}}*\boldsymbol{FD}_{\boldsymbol{m}}*\boldsymbol{FD}_{\boldsymbol{d}}*\boldsymbol{R} (\mathbf{1})$$

- Uncertainty estimation

| Source | Value | Standard_Unit | Unit | Sensitivity_Coefficient | Contribution |
| --- | --- | --- | --- | --- | --- |
| Calibrant 1 (Cn1) | 0.018 | 1.00E-03 | mg/kg | 150 - | 0.23% |
| Calibrant 1 Response (Rn1) | 0.8339 | 4.7 E-03 | UA | 3.5 mg/(kg*UA) | 5.60% |
| Calibrant 2 (Cn2) | 0.022 | 1.00E-03 | mg/kg | 47 - | 0.03% |
| Calibrant 2 Response (Rn2) | 0.994 | 7.6 E-3 | UA | 1.1 mg/(kg*UA) | 1.99% |
| Digestion Dilution (FDd) | 66.582 | 1.40E-04 | N/A | 0.057 mg/kg | 0.01% |
| Repeatability (R) | NA | 0.0136 | mg/kg | 3.8 N/A | 75.31% |
| Sample Dilution (FDm) | 2.9986 | 5.80E-05 | N/A | 1.3 mg/kg | 0.00% |
| Sample Response (Rm) | 0.8744 | 6.0 E-06 | UA | 4.6 mg/(kg*UA) | 16.83% |

GCMS Method.

- Model quantification

$$\boldsymbol{C}_{\boldsymbol{bracketing}}=\frac{\boldsymbol{C}_{\boldsymbol{n}\mathbf{2}}*\left( \frac{\boldsymbol{A}_{\boldsymbol{m}}}{\boldsymbol{A}_{\boldsymbol{ei} \boldsymbol{en} \boldsymbol{m}}}-\frac{\boldsymbol{A}_{\boldsymbol{n}\mathbf{1}}}{\boldsymbol{A}_{\boldsymbol{ei} \boldsymbol{en} \boldsymbol{n}\mathbf{1}}} \right)-\boldsymbol{C}_{\boldsymbol{n}\mathbf{1}}\left( \frac{\boldsymbol{A}_{\boldsymbol{m}}}{\boldsymbol{A}_{\boldsymbol{ei} \boldsymbol{en} \boldsymbol{m}}}-\frac{\boldsymbol{A}_{\boldsymbol{n}\mathbf{2}}}{\boldsymbol{A}_{\boldsymbol{ei} \boldsymbol{en} \boldsymbol{n}\mathbf{2}}} \right)}{\left( \frac{\boldsymbol{A}_{\boldsymbol{n}\mathbf{2}}}{\boldsymbol{A}_{\boldsymbol{ei} \boldsymbol{en} \boldsymbol{n}\mathbf{2}}}-\frac{\boldsymbol{A}_{\boldsymbol{n}\mathbf{1}}}{\boldsymbol{A}_{\boldsymbol{ei} \boldsymbol{en} \boldsymbol{n}\mathbf{1}}} \right)}*\boldsymbol{FD}_{\boldsymbol{m}}*\boldsymbol{R} (\mathbf{3})$$

- Uncertainty estimation

| Source | Value | Standard_Unit | Unit | Sensitivity_Coefficient | Contribution |
| --- | --- | --- | --- | --- | --- |
| Calibrant 1 (Cn1) | 0.47 | 1.73E-04 | mg/kg | 5.3 N/A | 0.00% |
| Calibrant 2 (Cn2) | 0.71 | 2.31E-04 | mg/kg | 1.7 N/A | 0.00% |
| Sample Dilution (FDm) | 7.06 | 0.029 | N/A | 0.53 mg/kg | 1.44% |
| Repeatability (R) | 1 | 0.0294 | mg/kg | 3.7 N/A | 16.44% |
| Sample Area (Am) | 27998 | 469 | Área | 2.80E-04 mg/(kg*Area) | 31.71% |
| Internal Standard Area  in Sample (Aei in m) | 13390 | 155 | Área | 5.80E-04 mg/(kg*Area) | 15.24% |
| Calibrant 1 Area (An1) | 22447 | 415 | Área | 2.50E-04 mg/(kg*Area) | 20.30% |
| Internal Standard Area  in Calibrant 1 (Aei in n1) | 11333 | 144 | Área | 4.90E-04 mg/(kg*Area) | 9.48% |
| Calibrant 2 Area (An2) | 18654 | 226 | Área | 1.20E-04 mg/(kg*Area) | 1.27% |
| Internal Standard Area  in Calibrant 2 (Aei in n2) | 7660 | 164 | Área | 2.90E-04 mg/(kg*Area) | 4.11% |
